# A neutralization assay for respiratory syncytial virus using a quantitative PCR-based endpoint assessment

**DOI:** 10.1186/1743-422X-10-195

**Published:** 2013-06-15

**Authors:** Jan C Varada, Belete Teferedegne, R Lynne Crim, Thembi Mdluli, Susette Audet, Keith Peden, Judy Beeler, Haruhiko Murata

**Affiliations:** 1 Laboratory of DNA Viruses, Division of Viral Products, OVRR, CBER, FDA, Bethesda, MD 20892, USA; 2 Laboratory of Pediatric and Respiratory Viral Diseases, Division of Viral Products, OVRR, CBER, FDA, Bethesda, MD 20892, USA; 3 Present affiliation: Weldon School of Biomedical Engineering, Purdue University, West Lafayette, IN, USA

**Keywords:** Respiratory syncytial virus, Neutralization, Antibody, Reverse transcription, RNA, Quantitative PCR, SYBR Green

## Abstract

**Background:**

Few studies have used quantitative polymerase chain reaction (qPCR) as an approach to measure virus neutralization assay endpoints. Its lack of use may not be surprising considering that sample nucleic acid extraction and purification can be expensive, labor-intensive, and rate-limiting.

**Methods:**

Virus/antibody mixtures were incubated for one hour at 37°C and then transferred to Vero cell monolayers in a 96-well plate format. At 24 (or 48) hours post-infection, we used a commercially available reagent to prepare cell lysates amenable to direct analysis by one-step SYBR Green quantitative reverse transcription PCR using primers specific for the RSV-N gene, thereby obviating the need for cumbersome RNA extraction and purification. The neutralization titer was defined as the reciprocal of the highest dilution needed to inhibit the PCR signal by 90% when compared with the mean value observed in virus control wells in the absence of neutralizing antibodies.

**Results:**

We have developed a qPCR-based neutralization assay for human respiratory syncytial virus. Due to the sensitivity of qPCR in detecting virus replication, endpoints may be assessed as early as 24 hours post-infection. In addition, the dynamic range of qPCR provides a basis for the assay to be relatively robust to perturbations in input virus dose (*i*.*e*., the assay is in compliance with the Percentage Law).

**Conclusions:**

This qPCR-based neutralization assay is suitable for automated high-throughput applications. In addition, our experimental approach may be generalizable for the rapid development of neutralization assays for other virus families.

## Background

Human respiratory syncytial virus (RSV), an enveloped RNA virus of the *Paramyxoviridae* family, is a pathogen of primary importance that can cause severe respiratory illness associated with high hospitalization rates and excess morbidity/mortality in susceptible populations such as infants, children, and the elderly [1-3]. RSV circulating among humans can be broadly categorized into two antigenic subgroups (A and B) [4]. The high prevalence of RSV results in most individuals being exposed as children within the first two years of life, and thereafter, recurrent infections can take place through adulthood [5]. Effective pharmacotherapy for RSV currently remains limited. The nucleoside analogue ribavirin is the only approved drug for RSV infection, but its clinical use is infrequent due to marginal efficacy [6]. A humanized monoclonal antibody with RSV-neutralizing activity is only licensed for prophylaxis in infants at high risk for severe RSV disease [7]. No vaccine is available for the prevention of RSV infection despite efforts spanning several decades [8-10]. Notable in the history of RSV vaccine development is the phenomenon of disease enhancement observed in recipients of a formalin-inactivated RSV vaccine formulation during clinical trials in the 1960’s [11-14]; this experience serves as a prominent example of the complexity that can be encountered during the course of vaccine development.

Serum neutralizing antibodies play an important role in conferring protection against RSV infection [7,15-17]. Traditional methods for measuring RSV-neutralizing activity in biological samples are labor-intensive and time-consuming. Plaque-reduction neutralization (PRN) entails numerous manipulation steps that hinder throughput, and plaque visualization can require several days [18]. Microneutralization assays for RSV using endpoint assessments based on ELISA [19], automated plaque counting [20,21], spectrophotometric quantification of cell viability [22], or enzymatic measurement of a reporter activity [23] require post-infection durations of 2–5 days. A recently developed neutralization assay for RSV based on using flow cytometry to evaluate infection by GFP-expressing RSV reporter viruses can measure the endpoint at 18 hours post-infection [24]; however, this assay requires a sophisticated instrument (a flow cytometer) that may preclude broad accessibility for interested investigators. Thus, a need still exists for a simple, rapid microneutralization assay suitable for high-throughput applications. Such an assay might be a useful tool to facilitate RSV vaccine development since one can anticipate the need to test thousands of samples to identify RSV susceptibles prior to immunization and to assess immune responses afterwards.

Quantitative PCR (qPCR) is associated with a number of appealing features, particularly in terms of robustness, sensitivity, and dynamic range. However, to date, few studies have used this experimental approach to quantify the extent of virus neutralization [25,26]. Normally, the need for RNA/DNA purification from samples represents a significant constraint that can decrease throughput in qPCR-based assays. We recently developed a qPCR-based neutralization assay for influenza virus by making use of a commercial reagent that allows the generation of PCR-ready cell lysates with minimal effort, thus circumventing a previously rate-limiting technical obstacle [27]. In the present study, we have exploited the sensitivity afforded by qPCR to develop a rapid 96-well format microneutralization assay for RSV with an assessment of endpoint as early as 24 hours post-infection. In addition, the dynamic range intrinsic to qPCR allows this assay to be relatively robust to perturbations in input virus dose. Considering the relative ease of generating experimental samples for analysis as well as the possibility for relying on automation to prepare qPCR plates, this assay might be appropriate for high-throughput purposes.

## Results

### qRT-PCR performance parameters

**Figure 1:**
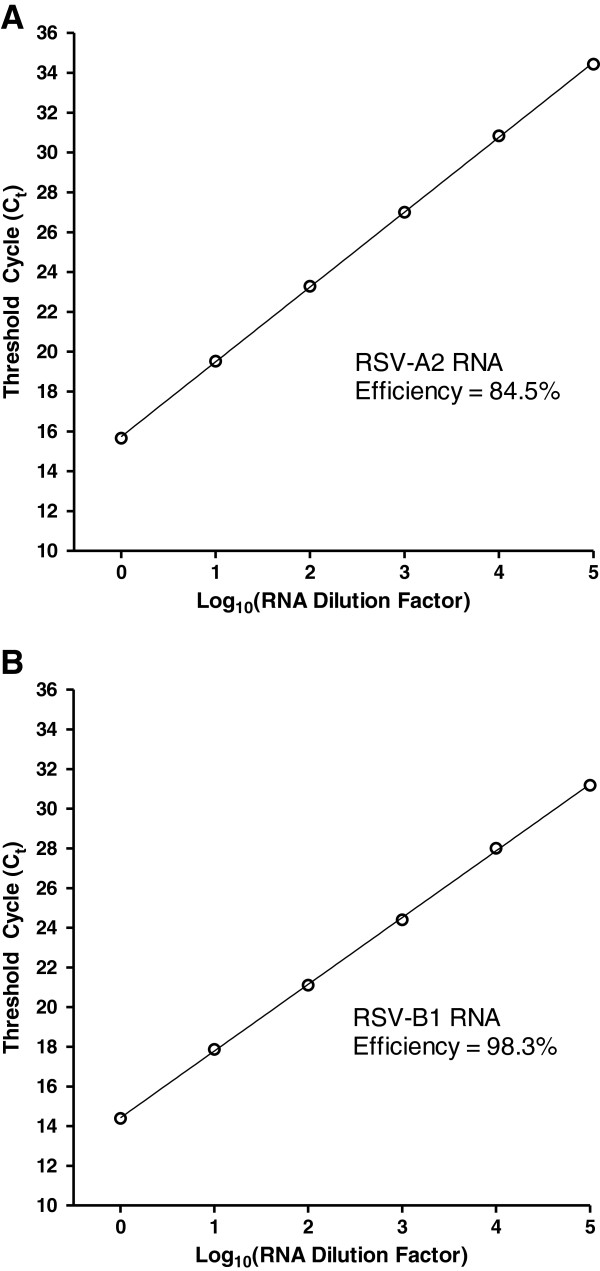
**SYBR Green qRT**-**PCR targeting the RSV N gene.** Total RNA was purified from Vero cells infected with either RSV-A2 or RSV-B1. The resulting RNA standards were serially diluted (10-fold); a cell lysate, prepared from uninfected Vero cells with the Bio-Rad iScript Sample Preparation Reagent (Bio-Rad SPR), was used as the diluent (the initial dilution contained ~10 ng/μL of RNA standard). One μL of each dilution was subjected to one-step SYBR Green qRT-PCR (10 μL total reaction volume). Mean threshold cycle (C_t_; n = 2) is plotted against log_10_ (RNA dilution factor) for each RNA standard dilution series: (**A**) RSV-A2 RNA and (**B**) RSV-B1 RNA.

Two pairs of SYBR Green qPCR primers, each targeting a conserved region of the N gene of RSV subgroup A or B [28], were used in our study. Purified total RNA standards from Vero cells infected with either RSV-A2 (subgroup A) or RSV-B1 (subgroup B) were prepared for the purpose of testing the performance features of our one-step quantitative reverse transcription SYBR Green PCR (qRT-PCR). In order to improve comparability with experimental samples, the purified RNA standards were serially diluted (10-fold) using a relevant matrix as the diluent. This matrix consisted of a lysate of uninfected Vero cells prepared using the Bio-Rad iScript Sample Preparation Reagent (subsequently referred to as Bio-Rad SPR). One μL of each dilution was subjected to one-step qRT-PCR in a total assay volume of 10 μL. An automated liquid-handling system was routinely used in the preparation of reactions for our study. Typical results are shown in Figures 1A (RSV-A2 RNA dilution series) and 1B (RSV-B1 RNA dilution series). PCR efficiency was ~85% for RSV subgroup A primers and ~98% for RSV subgroup B primers; for both primer pairs, linearity was observed over at least a 5 log_10_ range. RNA standards were used in subsequent experiments to verify the performance of each PCR run and to facilitate the quantification of experimental samples.

### Replication kinetics of RSV assessed by qRT-PCR

**Figure 2:**
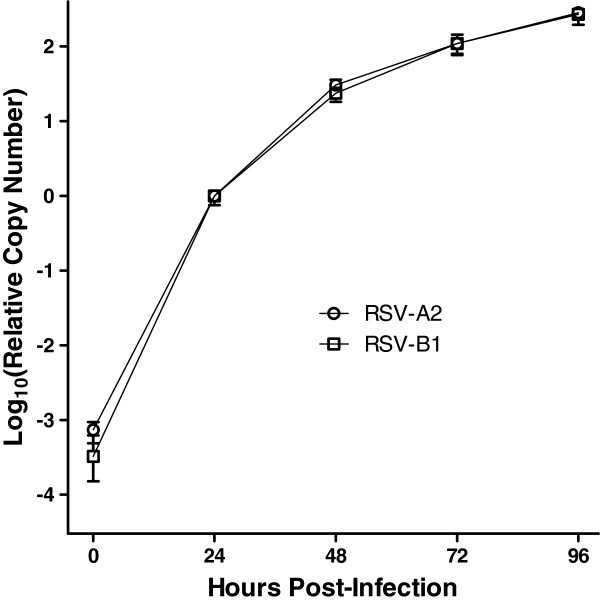
**Replication kinetics of RSV.** Vero cells were seeded in 96-well culture plates (15,000 cells per well). On the following day, cells were infected with RSV-A2 or RSV-B1 (500 TCID_50_ per well). After incubation for 1 hour at 37°C, the cells were washed twice with PBS to remove unbound virus; subsequently, the culture medium was replaced and the infection was allowed to proceed at 37°C. At the indicated times, cell lysates were prepared using Bio-Rad SPR and subjected to one-step SYBR Green qRT-PCR. RNA copy numbers were normalized to the mean value obtained at 24 hours post-infection for each virus strain. Each point represents the mean with corresponding range (n = 3).

Replication of RSV was assessed by qRT-PCR using cells infected in 96-well culture plates. Vero cells were seeded overnight (15,000 cells per well) and on the following day infected with 500 TCID_50_ per well of RSV-A2 or RSV-B1. After incubation for 1 hour at 37°C, the cells were washed twice with PBS (100 μL per well) to remove unbound virus; subsequently, the culture medium (200 μL per well) was replaced, and the infection was allowed to proceed at 37°C. Every 24 hours, cell lysates were prepared using Bio-Rad SPR according to the following simple steps: (1) remove the culture medium and wash the cells twice with 100 μL of PBS; (2) apply 100 μL of Bio-Rad SPR per well and incubate at room temperature for 1 min; and (3) collect the resulting lysate and freeze at −20°C until assessment by qRT-PCR. The replication kinetics of RSV-A2 and RSV-B1 are shown in Figure 2. RNA copy numbers were normalized to the mean value obtained 24 hours post-infection. For both RSV-A2 and RSV-B1 strains, the PCR signal increased by approximately 10^5^-fold over the course of 96 hours. In addition, an amplification of >1000-fold was observed for both virus strains over the initial 24 hours. The data support the feasibility of assessing virus neutralization at 24 hours post-infection.

### Correspondence of qPCR signal and input virus dose

**Figure 3:**
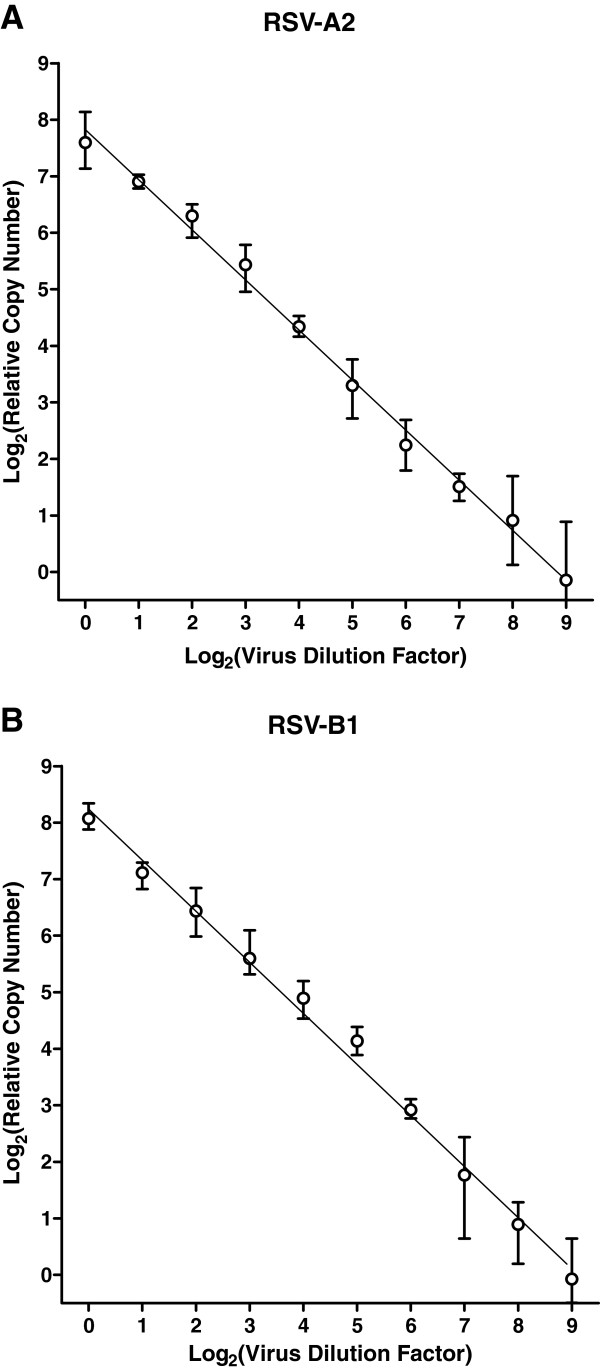
**qPCR signal *vs. *input virus dose.** Vero cells were seeded in 96-well culture plates (15,000 cells per well). On the following day, each well was infected with an inoculum from a two-fold dilution series: 40,000 to 78 TCID_50_ per well for (**A**) RSV-A2, and 250,000 to 490 TCID_50_ per well for (**B**) RSV-B1. At 24 hours post-infection, cell lysates were prepared using Bio-Rad SPR and subjected to qRT-PCR analysis. RNA copy numbers were normalized to the mean value obtained with the most dilute virus input. Each point represents the mean with corresponding range (n = 4).

The ability to distinguish two-fold differences in virus input was assessed. Vero cells were seeded overnight (15,000 cells per well), and on the following day, monolayers were infected with inocula derived from two-fold dilution series of RSV-A2 (40,000 to 78 TCID_50_ per well) or RSV-B1 (250,000 to 490 TCID_50_ per well). At 24 hours post-infection, cell lysates were prepared using Bio-Rad SPR and subjected to qRT-PCR analysis. RNA copy numbers were normalized to the mean value obtained with the most dilute input (78 TCID_50_ for RSV-A2 and 490 TCID_50_ for RSV-B1). Reasonable linearity was observed across the entire range of virus input for both RSV-A2 (Figure 3A) and RSV-B1 (Figure 3B). The data suggest that relatively small variations in infectivity (two-fold) can be measured with our approach.

### qRT-PCR-based microneutralization assay for RSV (qPCR-MN)

**Figure 4:**
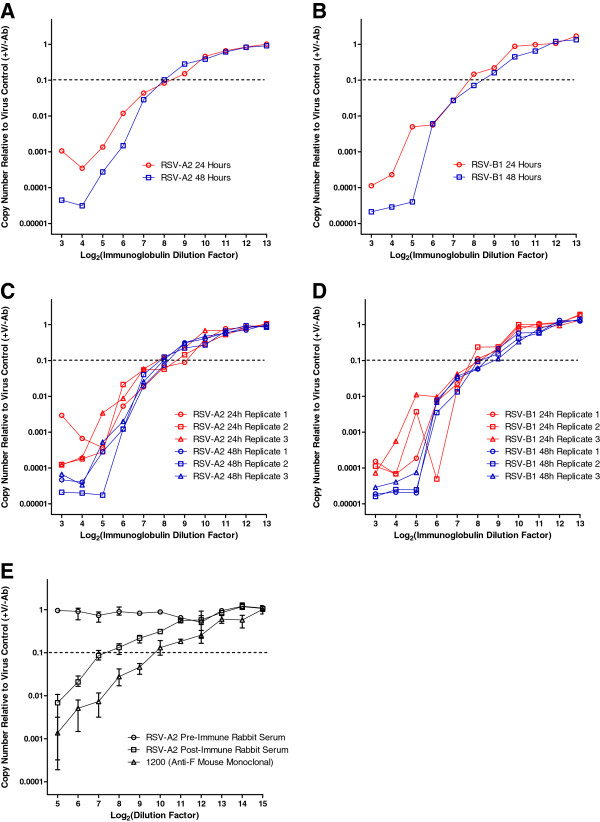
**qRT**-**PCR**-**based microneutralization of RSV (qPCR-MN).** Vero cells were seeded in 96-well culture plates (15,000 cells per well). On the following day, a two-fold dilution series was prepared from a pooled human immunoglobulin reference standard (designated as RSV-Lot 1) starting from an initial concentration of 1%. The virus inoculum (500 TCID_50_ per well of RSV-A2 or RSV-B1) was mixed with an equal volume of RSV-Lot 1 dilution and incubated for 1 hour at 37°C. After incubation, the mixture was transferred to the plate of seeded Vero cells. At 24 or 48 hours post-infection, cell lysates were prepared using Bio-Rad SPR and subjected to qRT-PCR analysis. RNA copy numbers were normalized to the mean value obtained from virus-infected control wells in the absence of neutralizing immunoglobulin. The neutralization titer was defined as the reciprocal of the highest dilution factor of RSV-Lot 1 necessary to inhibit the PCR signal by 90% (or below the threshold of 10% of the virus control wells indicated by the dotted line). (**A**) RSV-A2 neutralization assessed at 24 or 48 hours post-infection (each point represents the mean; n = 3). (**B**) RSV-B1 neutralization assessed at 24 or 48 hours post-infection (each point represents the mean; n = 3). The individual experimental replicates assessed independently (n = 3) are shown for neutralization experiments with (**C**) RSV-A2 and (**D**) RSV-B1. Additional neutralization experiments (**E**) with RSV-A2 assessed at 24 hours post-infection were also performed with a monoclonal antibody with known specificity to the RSV F protein (1200) as well as rabbit sera generated pre- and post-immunization with RSV-A2 (each point represents the mean with corresponding range; n = 3).

A qRT-PCR-based microneutralization assay for RSV (qPCR-MN) was performed to assess the neutralization activity associated with a pooled human immunoglobulin reference standard (designated as RSV-Lot 1) at a working concentration of 1% (1 mg/100 μL). Vero cells were seeded overnight (15,000 cells per well); on the following day, the virus inoculum (500 TCID_50_ per well of RSV-A2 or RSV-B1) was mixed with an equal volume from a two-fold dilution series of RSV-Lot 1 and incubated for 1 hour at 37°C. After incubation, the virus/immunoglobulin mixture was transferred to corresponding wells on a 96-well plate containing Vero cell monolayers. At 24 or 48 hours post-infection, cell lysates were prepared using Bio-Rad SPR and subjected to qRT-PCR analysis. The results are shown in Figure 4. RNA copy numbers were normalized to the mean value obtained from virus-infected wells in the absence of neutralizing immunoglobulin (virus control wells). Representative neutralization experiments consisting of three experimental replicates (reflecting three independent RSV-Lot 1 dilution series) are shown in Figures 4A (RSV-A2) and 4B (RSV-B1). The neutralization titer was defined as the reciprocal of the highest dilution factor of RSV-Lot 1 necessary to inhibit the PCR signal by 90% (*i*.*e*., below the threshold of 10% of the mean value observed in virus control wells). For both virus strains, the PCR signal decreased in a dose-dependent manner as the concentration of neutralizing antibodies increased; at maximal neutralization, the PCR signal was inhibited by >1000-fold. For both RSV-A2 and RSV-B1, similar results were obtained at 24 and 48 hours post-infection. Thus, while we have developed our assay with the intention of routinely assessing the neutralization endpoint at 24 hours post-infection, our data suggest that perturbations in assay duration can be tolerated to a certain extent (24 h *vs*. 48 h).

Each individual experimental replicate can be used to derive an independent estimate of the neutralization titer. The individual results of each replicate for the experiments shown in Figures 4A and 4B are shown in Figures 4C (RSV-A2) and 4D (RSV-B1); the mean of the curves depicted in Figure 4C would result in Figure 4A, while the mean of the curves depicted in Figure 4D would result in Figure 4B. For RSV-A2 neutralization, the geometric mean titer (GMT) calculated from experimental replicates for RSV-Lot 1 (1% concentration) was 156 assessed at 24 hours post-infection (7 experiments, each with 3 experimental replicates; n = 21) and 161 assessed at 48 hours post-infection (3 experiments, each with 3 experimental replicates; n = 9). Likewise for RSV-B1 neutralization, the GMT was 197 assessed at 24 hours post-infection (n = 21) and 299 assessed at 48 hours post-infection (n = 9). In comparison, the GMTs from plaque-reduction neutralization (PRN) for RSV-Lot 1 (at day 5 post-infection; assessed in HEp-2 cells for RSV-A2 or Vero cells for RSV-B1) in the absence of guinea pig complement were 238 and 450 against RSV-A2 and RSV-B1, respectively, while the PRN GMTs in the presence of guinea pig complement were 1176 and 2046 against RSV-A2 and RSV-B1, respectively.

Neutralization experiments with RSV-A2 assessed at 24 hours post-infection were also performed with a monoclonal antibody ascites fluid with known specificity to the RSV F protein (1200) [29] as well as rabbit serum samples generated pre- and post-immunization with UV-inactivated RSV-A2. The results are shown in Figure 4E. The GMTs (n = 3) were <32, 128, and 645 for pre-immune rabbit serum, post-immune rabbit serum, and monoclonal antibody 1200, respectively. The data suggest that the antibody-dependent decrease in signal observed in qPCR-MN reflects an inhibitory process with specificity against RSV.

### Compliance with the Percentage Law

**Figure 5:**
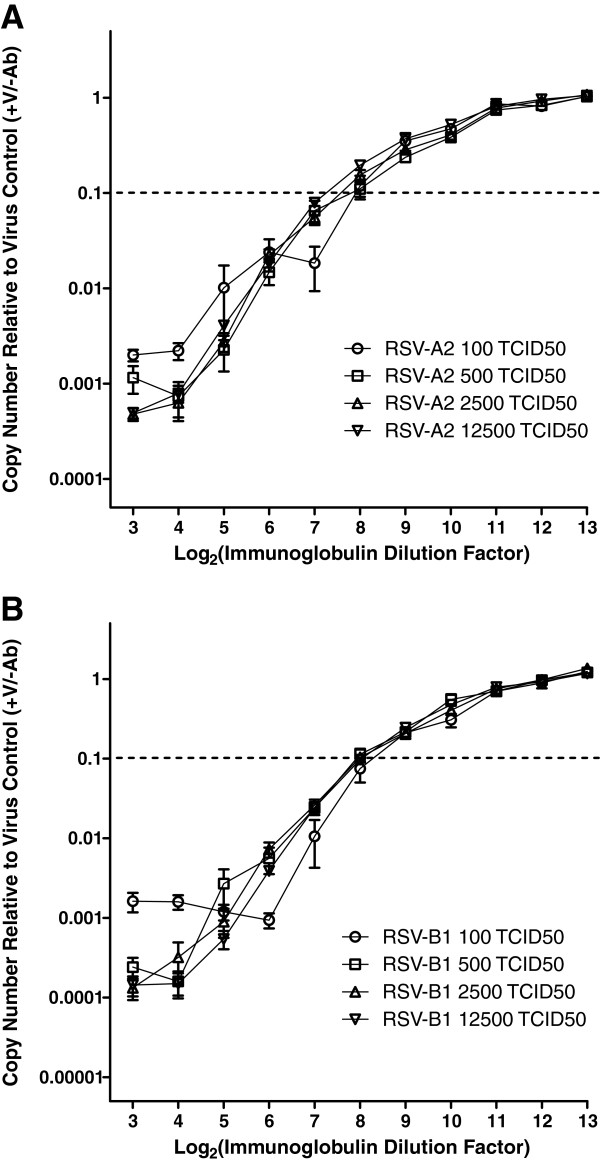
**Compliance with the Percentage Law.** Vero cells were seeded in 96-well culture plates (15,000 cells per well). On the following day, a two-fold dilution series was prepared from the human immunoglobulin standard (RSV-Lot 1) starting from an initial concentration of 1%. The virus inoculum (100, 500, 2500, 12500 TCID_50_ per well of RSV-A2 or RSV-B1) was mixed with an equal volume of RSV-Lot 1 dilution and incubated for 1 hour at 37°C. After incubation, the mixture was transferred to Vero cell monolayers. At 24 hours post-infection, cell lysates were prepared using Bio-Rad SPR and subjected to qRT-PCR analysis. RNA copy numbers were normalized to the mean value obtained from virus-infected control wells in the absence of neutralizing immunoglobulin. Each point represents the mean ± SEM from 3 experiments with 3 experimental replicates (n = 9) for (**A**) RSV-A2 neutralization and (**B**) RSV-B1 neutralization.

According to the Percentage Law [30,31], the proportion of infectivity neutralized by a given concentration of antibodies remains constant over a wide range of virus input, provided that the concentration of antibodies is in vast molar excess over the concentration of virus (a reasonable assumption under most experimental conditions). We assessed the compliance of our neutralization assay with the Percentage Law. Neutralization by pooled human immunoglobulin (RSV-Lot 1 at an initial concentration of 1%) was assessed at 24 hours post-infection using 100, 500, 2500, or 12500 TCID_50_ per well as virus inputs. The results are shown in Figures 5A (RSV-A2 neutralization) and 5B (RSV-B1 neutralization). For both RSV-A2 and RSV-B1, similar neutralization results were obtained regardless of input virus dose. The GMTs (3 experiments, each with 3 experimental replicates; n = 9) were 219, 149, 138, and 118 for RSV-A2 neutralization at 100, 500, 2500, or 12500 TCID_50_, respectively. The GMTs (n = 9) were 256, 174, 188, and 188 for RSV-B1 neutralization at 100, 500, 2500, or 12500 TCID_50_, respectively. Thus, qPCR-MN appears to be reasonably robust to perturbations in input virus across a >100-fold range, thereby demonstrating a performance feature predicted on the basis of the Percentage Law.

### Non-specific inhibition of RSV replication and neutralization assay limit of detection

**Figure 6:**
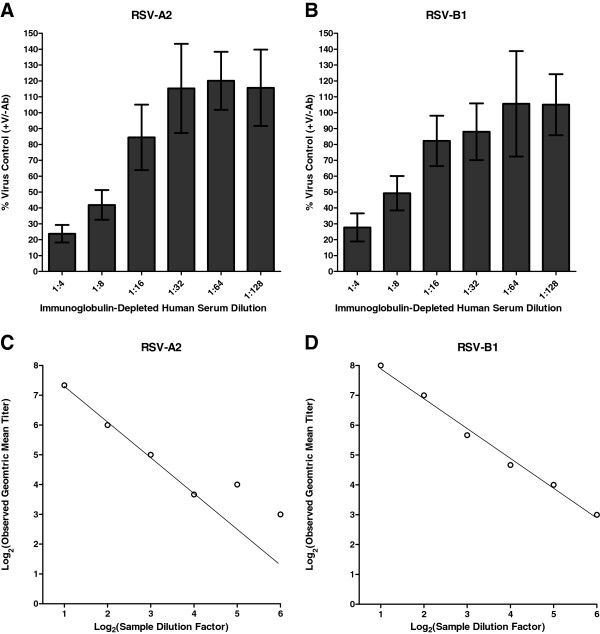
**Non**-**specific inhibition of RSV in minimally diluted samples.** Immunoglobulin-depleted human serum was assessed in qPCR-MN (Vero cells; 500 TCID_50_ per well of virus input; assessment at 24 hours post-infection) with (**A**) RSV-A2 and (**B**) RSV-B1. Values represent the mean ± SD (n = 9). Spiking experiments were also performed using the human immunoglobulin standard RSV-Lot 1. For the initial spiked sample, RSV-Lot 1 at 10% concentration was diluted 1:10 in immunoglobulin-depleted human serum. Five additional samples were generated by two-fold serial dilution using the depleted serum as the diluent. The resulting samples with simulated low neutralizing activity were assessed by qPCR-MN with (**C**) RSV-A2 and (**D**) RSV-B1. The observed GMT (n = 3) is plotted against the dilution factor of the simulated sample.

Non-specific inhibitory effects of human serum might limit the ability to detect low RSV-neutralizing activity. Immunoglobulin-depleted human serum was obtained from a commercial source and tested in our assay. The use of immunoglobulin-depleted human serum was necessary because of the difficulty in procuring serum samples from individuals truly naive to RSV exposure. The results are shown in Figures 6A (RSV-A2) and 6B (RSV-B1). For dilutions of depleted serum at or exceeding 1:16, marginal inhibition of virus replication was observed (PCR signal exceeded 80% of mean values observed in virus control wells). At higher concentrations of depleted serum, substantial inhibition of RSV replication was observed (PCR signal less than 30% of values observed in virus control wells for both RSV-A2 and RSV-B1 at a dilution of 1:4). Assuming that the tested immunoglobulin-depleted serum is representative of negative serum samples obtainable from an exposure-naive (hypothetical) population, routine assessment of a human serum sample might require an initial dilution of 1:16 or greater.

We sought to investigate the assay limit of detection further by analyzing samples generated by spiking the immunoglobulin-depleted human serum with the reference pooled human immunoglobulin RSV-Lot 1. For the initial spiked sample, RSV-Lot 1 at 10% concentration was diluted 1:10 in depleted serum. Five additional samples were generated by two-fold serial dilution using the depleted serum as the diluent. In this manner, we generated serum samples with simulated low neutralizing activity. The results of analyzing these samples are shown in Figures 6C (RSV-A2) and 6D (RSV-B1). The observed GMT (n = 3) is plotted against the dilution factor experienced by the simulated sample. For RSV-B1 (Figure 6D), a reasonably linear relationship was observed across the sample dilution range. Thus, the observed titer was generally in agreement with the expected titer. For RSV-A2 (Figure 6C), a linear relationship was observed until the sample experienced a dilution of 16-fold (observed GMT of 13); beyond this dilution, a departure from linearity was observed. The data suggest that, under some experimental conditions, non-specific inhibitory effects of the sample matrix might limit the ability to accurately detect low neutralizing activity.

### Correlation between titers measured by qPCR-MN and PRN

**Figure 7:**
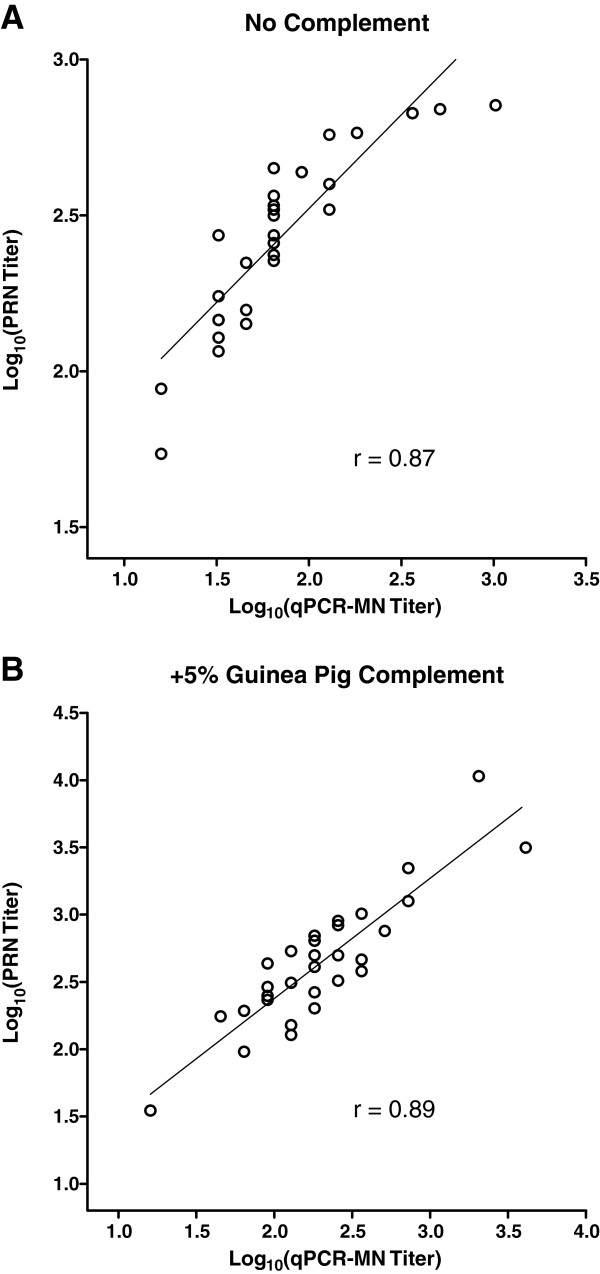
**Correlation between qPCR**-**MN and plaque**-**reduction neutralization (PRN).** Neutralization assays were performed in the (**A**) absence or (**B**) presence of 5% guinea pig complement with RSV-A2 using a panel of human serum samples (n = 30). PRN was performed using HEp-2 cells (50% inhibition endpoint). qPCR-MN (90% inhibition endpoint; geometric mean titers calculated from two experimental replicates) was performed using Vero cells (500 TCID_50_ per well of virus input; assessment at 24 hours post-infection).

We subjected archived human serum samples (n = 30; obtained from children who participated in a measles vaccine study in the 1980’s) to analysis by qPCR-MN and PRN in the absence or presence of 5% guinea pig complement. Neutralization against RSV-A2 was evaluated. PRN was performed using HEp-2 cells. For qPCR-MN (performed using Vero cells), 5% guinea pig complement was included in the neutralization reaction as necessary without altering other assay parameters or procedures. Final serum sample dilutions for qPCR-MN ranged from 1:32 to 1:8192; sera with qPCR-MN titers <32 (n = 2 in the absence of complement; n = 1 in the presence of complement) were assigned a titer of 16 for this analysis. The correlation coefficients were 0.87 (Figure 7A) and 0.89 (Figure 7B) in the absence or presence of guinea pig complement, respectively. The presence of guinea pig complement increased the observed titers for both assays. GMTs across samples (n = 30) were 268 and 440 for PRN (50% inhibition endpoint) in the absence or presence of guinea pig complement, respectively. Similarly, GMTs (n = 30) were 69 and 199 for qPCR-MN (90% inhibition endpoint) in the absence or presence of guinea pig complement, respectively.

## Discussion

We have developed a simple qRT-PCR-based microneutralization assay for RSV (qPCR-MN). The crux of our approach is the use of a commercially available reagent (Bio-Rad SPR) to generate cell lysates that are amenable to direct analysis by qRT-PCR, thereby allowing us to avoid the need for RNA extraction and purification (which normally can be laborious and rate-limiting). Certain features intrinsic to qPCR offer significant advantages in the context of our study. The sensitive detection of RSV replication allows our qPCR-MN neutralization endpoint to be assessed at 24 hours post-infection (and likely earlier). In addition to time-saving benefits, a shorter assay duration is preferable *a priori* when assessing the neutralization of replication-competent viruses because it minimizes possible distortions associated with multiple cycles of virus replication (as discussed in our earlier study on influenza virus neutralization [27]). The dynamic range of qRT-PCR (at least 5 log_10_ range; Figure 1) directly contributes to qPCR-MN being relatively robust to perturbations in input virus dose and allows the empirical demonstration of compliance with the Percentage Law (Figure 5) without the need to dilute or otherwise manipulate reactions in order to quantify the extent of virus neutralization. Other logistically desirable attributes of qPCR-MN include the feasibility of incorporating automation for the preparation of qRT-PCR and the ease of standardization across independent laboratories through dissemination of frozen RNA reagents and samples.

We also wish to emphasize the flexibility of our approach. We generated data for our study using two virus strains (RSV-A2 and RSV-B1). However, the qPCR primer pairs used in our study (targeting the conserved N gene) should be immediately applicable to a diverse range of virus strains and field isolates without the need for major adjustments in assay parameters. The performance of a nucleic acid-based assay targeting a conserved region of the viral genome might be more predictable across a wider range of strains than assays relying on other RSV-specific detection reagents such as antibodies. As an example, the monoclonal antibody A3, used as a detection reagent in ELISA-based microneutralization assays for influenza virus, exhibits a range in its sensitivity to detect the nucleoprotein (NP) from influenza A viruses that varies over at least two orders of magnitude despite the high degree of conservation of NP [32]. Thus, qPCR-MN might be a useful tool to dissect subtle antigenic differences within the subgroups of RSV. In addition, although we primarily used Vero cells to generate data for our study, our approach does not preclude the use of other cell lines. For example, we were successful in performing qPCR-MN for RSV using HEp-2 cells (data not shown). The suitability of a cell line for qPCR-MN can be dictated by circumstances particular to a given experiment (for example, the growth property of the RSV strain).

The current cost (~$1 per qRT-PCR well) associated with qPCR-MN (mainly due to Bio-Rad SPR as well as the commercial kit for one-step qRT-PCR) might be acceptable for many purposes, even ones requiring high sample throughput, especially considering the relatively minimal effort necessary for generating suitable experimental samples for qRT-PCR analysis and the possibility for exploiting automation. For example, screening serum samples using a single dilution to identify RSV susceptible subjects for inclusion in clinical trials might be feasible with this type of assay since only a small volume of serum is required and since the assay potentially provides a rapid determination of serostatus. We are actively investigating additional cost-cutting measures including further downscaling and substitution of key reagents associated with substantial expense (*i*.*e*., Bio-Rad SPR) with less costly alternatives.

We have used a 90% inhibition endpoint for qPCR-MN in the present study. A 50% inhibition endpoint resulted in greater variability in neutralization titer estimation among replicates compared with the use of a 90% inhibition endpoint. Other endpoint definitions (for example, a 50% inhibition endpoint calculated according to a non-parametric algorithm based on the method of Spearman and Kärber) could be used, but the correlation of results with PRN titers (using a 50% inhibition endpoint) marginally decreased (data not shown). In future studies, the endpoint definition for qPCR-MN might need to be refined using larger sample sizes from diverse populations in order to identify a biologically relevant neutralization threshold that accurately discriminates between RSV-susceptible and RSV-protected individuals. It is important to note that there may be intrinsic differences in the functional activity of the antibodies evaluated in an assay that measures 90% inhibition within a single virus infection cycle as compared with an assay that measures 50% reduction in plaque counts following multiple cycles of virus replication. These qualitative differences could contribute to the minor differences in results observed between qPCR-MN and PRN.

## Conclusions

We have developed a simple qRT-PCR-based microneutralization assay (qPCR-MN) for RSV amenable to high-throughput applications. Our approach relies upon a commercially available reagent that circumvents sample RNA purification, thereby removing a procedural obstacle that can be labor-intensive and rate-limiting. Reagents that require the expenditure of substantial time or resources to generate (reporter virus constructs for different strains, antibodies, etc.) are not necessary for qPCR-MN. The sensitivity and dynamic range of qRT-PCR can be exploited to allow neutralization endpoint assessment at 24 hours post-infection as well as empirical demonstration of compliance with the Percentage Law (*i*.*e*., robustness to perturbations in input virus dose). Other appealing features of qPCR-MN include the possibility for incorporation of automation and the availability of the option to disseminate frozen RNA reagents and samples for the purpose of assay standardization. Finally, we note that our qRT-PCR-based approach might be generalizable for the rapid development of neutralization assays involving other families of viruses.

## Methods

### Viruses, cells, and antibodies

RSV-A2, a gift from Robert Chanock and Brian Murphy (NIAID, NIH, USA), was serially plaque-purified 3 times and then amplified in HEp-2 cells to generate the working virus pool. RSV-B1, a gift from Fran Rubin (NIAID, NIH), was amplified in Vero cells after receipt to generate the working virus pool. HEp-2 and Vero cells (obtained from ATCC; Manassas, VA, USA) were grown in Eagle’s MEM supplemented with 4 mM glutamine and 10% fetal bovine serum (MEM-10); RSV infections were performed with MEM supplemented with 4 mM glutamine and 1% fetal bovine serum (MEM-1) as described [33]. Extracellular virus was harvested when the observed cytopathic effect (CPE) reached 4+ (72 to 120 hours after infection; MOI of 0.1), clarified by low-speed centrifugation and stabilized by addition of 100 mM MgSO_4_ and 50 mM HEPES buffer, and stored at −70°C in aliquots until use. For animal immunizations, RSV-A2 was purified using a 30/45/60% discontinuous sucrose gradient with ultracentrifugation at 35K RPM in a Beckman SW41 rotor for 2 hours at 4°C. The purified virus fraction was inactivated by exposure to ultraviolet light (254 nm) for 30 minutes as described [34], and inactivation was confirmed by absence of CPE following incubation on Vero cell monolayers for 6 days.

A lot of human immunoglobulin (intended for intravenous human administration) was obtained from Baxter (Deerfield, IL, USA). Following extensive characterization (J. Beeler; manuscript in preparation), this lot was designated as “RSV-Lot 1” to be used as a reference reagent. RSV-F monoclonal antibody 1200 (ascites fluid) with known neutralizing activity was generated and characterized as previously described [29]. Rabbit anti-RSV polyclonal sera were prepared by immunizing New Zealand white rabbits subcutaneously with a dose equivalent of 10^6^ TCID_50_ of UV-inactivated, purified RSV-A2 in complete Freund’s adjuvant; rabbits were boosted two times with the same dose in incomplete Freund’s adjuvant every three weeks. This protocol was approved by the CBER Institutional Animal Care and Use Committee (IACUC; FDA, USA). Pre-immune sera were collected before the first immunization, and post-immune sera were collected four weeks after the last boost. Archived, de-identified human serum samples from children in the 6-8^th^ grades who participated in a measles vaccine study in the 1980’s were assessed following approval from the Research Involving Human Subjects Committee (RIHSC; FDA, USA). Human serum depleted of IgG, IgM, and IgA was obtained from SunnyLab (SF142-2; Sittingbourne, UK). For certain neutralization experiments, the antibody samples were assessed in the presence of guinea pig complement (C200-0005; Rockland Immunochemicals, Inc.; Gilbertsville, PA, USA) at 5% in the final virus/antibody mixture (complement/antibody sample dilution mixed with an equal volume of virus).

### qRT-PCR-based microneutralization assay (qPCR-MN) for RSV

RNA standards were prepared by purifying total RNA (using the Qiagen RNeasy kit; Valencia, CA, USA) from Vero cells infected with RSV-A2 or RSV-B1 (MOI = 0.1; 72 hours post-infection).

For qPCR-MN, 96-well culture plates were seeded with Vero cells (15,000 cells per well). On the following day, the cell propagation medium (MEM-10) was replaced with the infection medium (MEM-1; 100 μL/well). Virus inocula were mixed with an equal volume of antibody dilutions (from a dilution series using MEM-1 as the diluent) and incubated at 37°C for 1 h; subsequently, 100 μL of the virus/antibody mixtures (containing a pre-neutralization infectivity of 500 TCID_50_ of RSV-A2 or RSV-B1) were transferred to the corresponding wells containing cell monolayers. At 24 h post-infection, experimental samples were prepared using the iScript Sample Preparation Reagent (Bio-Rad; Hercules, CA, USA; referred to as Bio-Rad SPR) by (1) removing the medium and washing the cells twice with 100 μL of PBS; (2) applying 100 μL of Bio-Rad SPR per well and incubating at room temperature for 1 min; and (3) collecting the resulting lysate. Sample lysates were routinely stored frozen at −20°C until assessment by qRT-PCR.

The following two primer pairs (targeting the RSV N gene) were used for qRT-PCR: CATCCAGCAAATACACCATCCA and TTCTGCACATCATAATTAGGAGTATCAA for RSV subgroup A viruses, and CTGTCATCCAGCAAATACACTATTCA and GCACATCATAATTGGGAGTGTCA for RSV subgroup B viruses [28]. Following optimization (performed as described previously [35]), reactions contained: each primer at 300 nM, 1X iScript One-Step SYBR Green RT-PCR mix (Bio-Rad), 1 μL of sample (Bio-Rad SPR lysate), and water to 10 μL. Reaction preparations were routinely performed using the epMotion 5075 automated liquid handling system (Eppendorf North America; Hauppage, NY, USA). Real-time PCR was performed using the CFX-96 instrument (Bio-Rad) using the following conditions: 50°C for 10 min (1X)/ 95°C for 5 min (1X)/ 95°C for 10 s, 63°C (for RSV subgroup A primers) or 64°C (for RSV subgroup B primers) for 30 s (40X). The RNA standards (serially diluted in a Bio-Rad SPR lysate prepared from uninfected Vero cells) were used to verify the performance of each PCR run and to facilitate the quantification of experimental samples. The RNA copy numbers were normalized to the mean value (n ≥ 3) obtained from virus-infected control wells in the absence of neutralizing antibodies (virus control wells). The neutralization titer was defined as the reciprocal of the highest dilution factor (after accounting for addition of virus) necessary to inhibit the PCR signal 90% (*i*.*e*., below the threshold of 10% of the mean value observed in virus control wells). Certain assay parameters (for example, input virus dose and incubation time) were altered as indicated without other assay adjustments.

### Plaque-reduction neutralization assay (PRN) for RSV

Plaque-reduction neutralization assays (PRN) were performed as previously described [18]. Human serum samples were heat inactivated at 56°C for 30 minutes prior to testing. Briefly, serial four-fold dilutions of antibody samples were incubated with RSV-A2 or RSV-B1 in the presence or absence of 5% guinea pig complement (diluted to yield 25–35 plaques per well) for 1 hour prior to inoculating virus/antibody mixtures onto HEp-2 cell monolayers in duplicate in 24-well cell culture plates for one hour at 37°C prior to overlaying with medium containing 1.5% methylcellulose. After 5 days, monolayers were fixed using 80% methanol and subjected to immunostaining with a mixture of two RSV-F monoclonal antibodies (1129 and 1243) [29]. Plates were then washed 3 times with PBS followed by addition of peroxidase-conjugated, goat anti-mouse antibody for 1 hour at 37°C. Plates were washed, treated with 100 μL of 4-chloro-1-naphthol in methanol (3 mg/mL) and 0.01% hydrogen peroxide until plaques were obvious to the eye with minimal background. Endpoint titers were calculated using the Spearman-Kärber method [36] and reported as the reciprocal of the dilution (after accounting for addition of virus) that decreased the plaque count by at least 50%.

## Ethical approval

The animal protocol was approved by the CBER Institutional Animal Care and Use Committee (IACUC; FDA, USA). The use of human serum samples was approved by the Research Involving Human Subjects Committee (RIHSC; FDA, USA).

## Competing interests

The authors declare that they have no competing interests.

## Authors’ contributions

JB, BT, KP, and HM were responsible for design and conception of the study. JV, BT, and HM performed the qPCR-based neutralization assay. TM, SA, LC, and JB performed the plaque-reduction neutralization analysis. JB, KP, and HM prepared the manuscript. All authors approved the final version of the manuscript.
